# Risk Factors for Delayed Bleeding and Its Impact on Long‐Term Efficacy After Endoscopic Sclerotherapy for Internal Haemorrhoids by Inverted Colonoscopy Without Transparent Caps

**DOI:** 10.1155/grp/1777665

**Published:** 2026-04-17

**Authors:** Yue Chen, Mingqiong Wang, Xing Su, Yuxia Zhu, Qin He, Mingke Li, Peng Zou, Chang Liu, Hongmei Liu, Ruizheng Zou

**Affiliations:** ^1^ Department of Gastroenterology, Liangjiang Hospital of Chongqing Medical University, People′s Hospital of Chongqing Liangjiang New Area, Chongqing, China; ^2^ Department of Nuclear Medicine, Liangjiang Hospital of Chongqing Medical University, People′s Hospital of Chongqing Liangjiang New Area, Chongqing, China

**Keywords:** delayed haemorrhage, endoscopy, haemorrhoids, lauromacrogol, sclerotherapy

## Abstract

**Background:**

We investigated the incidence, timing, risk factors and prognosis of delayed haemorrhage after endoscopic injection sclerotherapy (EIS) with lauromacrogol for internal haemorrhoids (IHs) using an inverted colonoscope without transparent caps.

**Methods:**

The clinical data of 252 patients undergoing EIS with lauromacrogol for IH using an inverted colonoscope without transparent caps were retrospectively analysed. Delayed haemorrhage was defined as bleeding occurring between 24 h and 1 month postoperatively. The incidence, timing and volume of delayed bleeding were recorded. Clinical risk factors were analysed, and patients were followed up for 2 years.

**Results:**

Delayed bleeding occurred in 17.5% (44/252) of patients, with a median onset of 2 (1–17) days. Among them, 97.7% (43/44) experienced < 20‐mL bleeding within 9 days that resolved spontaneously; one patient developed 500‐mL bleeding on postoperative Day 17. Multivariate logistic regression analysis showed that albumin < 40 g/L (odds ratio [OR]: 5.093; *p* < 0.001), triglycerides > 1.7 mmol/L (OR: 3.814, *p* < 0.001), Wexner Constipation Score ≥ 15 (OR: 5.340, *p* < 0.001) and > 4 injection sites (OR: 4.425, *p* = 0.005) were independent risk factors. The case of 500‐mL bleeding may have resulted from an excessively deep injection and a high injection position. After 2 years, treatment effectiveness did not differ significantly between patients with and without delayed bleeding (*p* = 0.622).

**Conclusions:**

Delayed bleeding is a common complication after EIS with lauromacrogol for IHs using an inverted colonoscope without transparent caps. Most cases are small volume, early onset and self‐limiting. Delayed bleeding does not affect long‐term EIS efficacy. Risk factors include hypertriglyceridaemia, hypoproteinaemia, postoperative constipation and > 4 injection sites. As delayed massive bleeding may occur when injections are too deep or positioned too high, EIS should be performed cautiously. Adherence to guidelines, including the use of a transparent cap or forward‐view endoscopy, is recommended.

## 1. Introduction

The incidence of haemorrhoids is high and increases with age, imposing a substantial economic burden [1, 2]. Internal haemorrhoids (IHs) can cause bleeding, swelling, discharge and other symptoms that significantly affect quality of life; in severe cases, excessive bleeding may be life‐threatening [3]. At present, the main treatment approaches for IH include conservative, surgical and instrumental modalities [4]. Conservative treatment is suitable only for patients with mild symptoms; however, it provides partial symptom relief, and recurrence is common [5]. Surgical treatment is invasive and associated with considerable postoperative pain, a higher rate of procedure‐related complications and prolonged recovery time, which may reduce patient acceptance. Instrumental treatments mainly include injection sclerotherapy, rubber band ligation and infrared photocoagulation [6].

Endoscopic injection sclerotherapy (EIS) for IH has attracted increasing attention owing to its minimal invasiveness, rapid recovery, low cost and relatively low complication rate [7]. EIS involves the injection of a sclerosing agent into the submucosal layer of the haemorrhoid, allowing the microvasculature of the IH to be exposed to the sclerosant and subsequently inducing fibrosis. Previous studies have demonstrated that EIS is associated with less pain and lower complication and recurrence rates compared with ligation therapy [8]. One of the most commonly used sclerosing agents is lauromacrogol [9].

Our hospital has performed EIS for IH using lauromacrogol. Prior to sclerotherapy, all patients underwent a complete colonoscopy using a standard colonoscope. EIS was subsequently performed with an inverted colonoscope to improve visualisation of the haemorrhoidal structures. To facilitate flexible inversion of the colonoscope and minimise tissue trauma, a transparent cap was not used.

Of note, our department operated with limited staffing resources relative to patient volume. This approach reflected constraints in personnel and equipment availability. In our practice, delayed bleeding was observed in some patients, particularly during the initial phase of implementing this treatment. Few studies have reported the specific clinical characteristics and risk factors associated with this complication. To provide evidence for clinical practice, we aimed to investigate the clinical features and risk factors of delayed haemorrhage following EIS with lauromacrogol for IH performed using an inverted colonoscope without transparent caps. In addition, we sought to evaluate the impact of delayed bleeding on the long‐term efficacy of the procedure.

## 2. Materials and Methods

### 2.1. Patient Selection

This retrospective study was conducted at the People′s Hospital of Chongqing Liangjiang New Area, Chongiqng, China. A total of 252 patients who underwent EIS with lauromacrogol for IH at our hospital between January 2021 and April 2022 were included in the analysis. All patients provided written informed consent prior to undergoing EIS for IH and were subsequently informed of their potential inclusion in this retrospective study. The study was approved by the Ethics Committee of the People′s Hospital of Chongqing Liangjiang New Area (Approval No. [19] of the Ethics Review Committee in 2023).

### 2.2. Inclusion and Exclusion Criteria

#### 2.2.1. Inclusion Criteria

The inclusion criteria were as follows: (a) Goligher Grade I–III IH with associated symptoms, including bleeding, prolapse, swelling and mucous discharge, and inadequate response to dietary or medical treatment; (b) age ≥ 18 years; (c) provision of informed consent and (d) availability of complete clinical data.

#### 2.2.2. Exclusion Criteria

The exclusion criteria were as follows: (a) severe cardiac, pulmonary, hepatic or renal insufficiency, or malignancy; (b) haematological disorders, psychiatric disorders or impaired consciousness; (c) use of antiplatelet or anticoagulant agents within 3 months prior to the procedure; (d) concomitant colonoscopic treatments rendering the source of bleeding indistinguishable and (e) IH complicated by incarceration, thrombosis, ulceration or infection.

### 2.3. EIS Procedure

#### 2.3.1. Bowel Preparation

Patients followed a low‐residue diet 1 day before colonoscopy and fasted for > 8 h. Bowel preparation consisted of a modified 3‐L polyethylene glycol electrolyte solution, with 1 L taken on the evening before the procedure and the remaining 2 L taken 4–6 h prior to the procedure.

#### 2.3.2. Colonoscopy

Patients were placed in the left lateral decubitus position with the hips and knees flexed. Total intravenous anaesthesia was administered using propofol and fentanyl. Following induction of anaesthesia, a lubricated endoscope (Olympus GIF‐Q260J or Fujifilm EC‐L600ZP7), with ultrasound gel (YY0299‐2016; Tianjin Grand Paper Industry Co. Ltd, Tianjin, China), was inserted into the anus. A complete colonoscopic examination was then performed.

#### 2.3.3. IH Exposure

To facilitate the procedure, we did not switch from colonoscopy to gastroscopy; instead, EIS was performed using a colonoscope. The procedure was conducted with an inverted colonoscope to better expose the haemorrhoidal structures [10]. To allow flexible inversion of the colonoscope and minimise tissue trauma, a transparent cap was not used. It should be noted that our department operated with limited staff and equipment resources relative to patient volume.

#### 2.3.4. EIS Procedure

After exposure of the IH and dentate line under an inverted colonoscope, a solution of lauromacrogol (10 mL/100 mg; code: 2021010703, Shaanxi Tianyu Pharmaceutical Co. Ltd, Shaanxi, China) mixed with methylene blue (1 mL/10 mg; code: 2105022, Jumpcan Pharmaceutical Group Co. Ltd, Taixing City, China) was injected into the core of the haemorrhoids using an injection needle. We elected to use 5‐mm short needles (model: VDK‐IN‐23‐230‐2304‐A; code: 2021011121IN, Jiangsu Vedkang Medical Science and Technology Co. Ltd, Jiangsu, China) to minimise the risk of excessively deep injection [10].

The injection site was located between the dentate line and the anorectal junction. The injection angle ranged from 30° to 45° [11, 12]. The single injection volume was ≤ 2 mL, and the total injection volume was ≤ 10 mL [11, 12]. All procedures were performed by our team of physicians experienced in endoscopic treatment.

**FIGURE 1:**
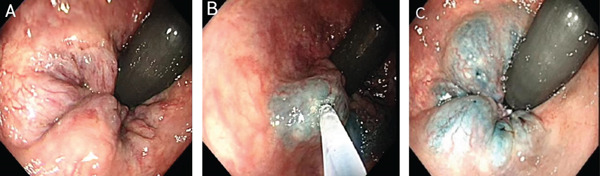
Endoscopic injection sclerotherapy with lauromacrogol for internal haemorrhoids. **(A)** Pretreatment. Internal haemorrhoids and the dentate line are visualised after reversed endoscopy. **(B)** During treatment. An injection needle is used to deliver lauromacrogol mixed with methylene blue into the core of the haemorrhoids. **(C)** Post‐treatment. The four injection points show uniform colour and good dispersion.

Postoperatively, patients were instructed to refrain from eating for 24–48 h, maintain perianal hygiene, monitor bowel movements and observe for the presence or absence of anal bleeding. The procedure for EIS with lauromacrogol for IH is illustrated in Figure 1.

### 2.4. Perioperative Medication

Some patients received intravenous or topical antibiotics, diosmin, carbazochrome sodium sulfonate or indomethacin–furazolidone suppositories during the perioperative period. These medications were primarily administered during the early stages of our treatment programme. With accumulated experience and adherence to guideline‐based protocols, the use of these agents progressively decreased. To determine whether these medications were associated with delayed bleeding, they were incorporated as potential influencing factors in our analysis. Our rationale for selecting the aforementioned drugs is as follows:

#### 2.4.1. Antibiotics

The anorectal region is a contaminated area. The injection site of sclerotherapy provides a potential pathway for bacterial invasion [13]. To prevent postoperative infection, intravenous antibiotics were administered during the early phase of our treatment programme, based on the assessment of the immune status of the patient by the physician and the recommendations of the operator. The 2025 expert consensus states that antibiotics may be used according to the immune status of the patient [10]. If preoperative white blood cell counts were elevated, suggesting infection, antibiotics were administered to control infection prior to EIS.

#### 2.4.2. Indomethacin–Furazolidone Suppositories

EIS may cause anal swelling and pain, which are common postoperative symptoms [14]. To alleviate such discomfort, indomethacin–furazolidone suppositories were introduced. This rectally administered formulation, approved in China, contains a combination of indomethacin and furazolidone and is indicated for haemorrhoids and postoperative anal and rectal pain relief. Suppositories are characterised by rapid onset, high local drug concentration and fewer systemic adverse effects [15]. Rectal suppositories are widely used for postoperative pain management, particularly following anorectal surgery [16].

Indomethacin inhibits cyclooxygenase‐2 activity, reduces prostaglandin synthesis and provides anti‐inflammatory and analgesic effects [17]. Furazolidone effectively inhibits *Escherichia coli* [18]. The suppository therefore provides local antibacterial action, potentially reducing the risk of postoperative infection and limiting the need for intravenous antibiotics.

#### 2.4.3. Diosmin

Reviews indicate that venoactive agents, such as micronised purified flavonoid fraction (MPFF), are used in the treatment of IHs. In acute haemorrhoidal disease, MPFF alleviates anal symptoms including bleeding, pain and discomfort. In patients undergoing surgery, postoperative adjunctive MPFF reportedly reduces pain and bleeding [19]. A longitudinal cohort study reported that combination therapy with MPFF and rubber band ligation significantly reduced bleeding intensity in the first month and itching during the first week [20].

MPFF is a venoactive agent that increases venous tone, reduces venous stasis, inhibits inflammatory mediators and enhances lymphatic drainage [21]. The intravenous agent available in our hospital is diosmin. Reportedly, diosmin can safely and effectively alleviate symptoms of IHs, including pain, discomfort, itching and bleeding, with a favourable safety profile [22]. When patients experience anal swelling, pain or discomfort before or after treatment, oral diosmin may be administered.

#### 2.4.4. Carbazochrome Sodium Sulfonate

Carbazochrome sodium sulfonate exerts rapid and sustained haemostatic effects without increasing thrombotic risk by constricting damaged capillaries, reducing vascular permeability and enhancing coagulation efficiency [23]. It has been used in orthopaedic postoperative bleeding, emergency haemorrhage, gynaecological bleeding, gastrointestinal bleeding, respiratory tract haemorrhage and haematuria [24–27]. Moreover, it alleviates anal discomfort and pain in patients with haemorrhoids [28].

Combination therapy with carbazochrome and parenteral troxerutin reportedly alleviates pain, discharge, bleeding, inflammation and pruritus in patients who underwent haemorrhoidectomy [29]. However, studies evaluating carbazochrome sodium sulfonate for colonic diverticular haemorrhage and postoperative bleeding following colonic endoscopic submucosal dissection have concluded that its haemostatic efficacy in these settings remains inconclusive [30, 31].

In our study, some patients presented with gastrointestinal bleeding symptoms, and carbazochrome sodium sulfonate was administered for haemostasis. To investigate whether its use influenced delayed bleeding after endoscopic haemorrhoid sclerotherapy, we included this variable in our analysis.

### 2.5. Observation Indicators and Follow‐Up

#### 2.5.1. Clinical Factors

The following data were collected: general characteristics, including age, sex and comorbidities, obtained from admission records; preoperative laboratory results, including routine blood tests, coagulation profiles and biochemical parameters, completed on admission; intraoperative haemorrhoid‐related variables, including grade, number of injection sites and total injection volume, as documented in the endoscopy report; postoperative fasting duration; and perioperative treatments, including carbazochrome sodium sulfonate (80 mg intravenously once daily), cefuroxime sodium (0.75 g intravenously twice daily), levofloxacin (0.5 g intravenously once daily), indomethacin–furazolidone suppositories (75 mg/100 mg rectally once daily) and diosmin (0.45 g orally twice daily), as recorded in physician orders.

The Wexner Constipation Score (WCS) was assessed. This score was calculated based on the following components: frequency of bowel movements, painful evacuation, incomplete evacuation, abdominal pain, duration of each attempt, need for assistance during evacuation, unsuccessful evacuation attempts within 24 h and duration of constipation, according to the condition of the patient within 1 month after treatment [32].

#### 2.5.2. Neglected Factors

In theory, procedural variables, such as injection angle, depth, position and single injection volume, should have been included as analytical factors. However, these parameters were not incorporated because they are difficult to quantify objectively. This represents a limitation of the present study.

#### 2.5.3. Definition of Delayed Bleeding

Delayed bleeding was defined as (1) absence of visible intraoperative bleeding followed by procedure‐related bleeding occurring between 24 h and 30 days postoperatively, manifested as blood on wiping, dripping blood after defaecation or bloody stools; or (2) intraoperative bleeding that was successfully controlled, followed by recurrent bleeding within 24 h to 30 days after the procedure.

#### 2.5.4. Assessment of Bleeding Volume

The volume of bleeding was estimated by comparison with common everyday objects as reference standards: 1.5 mL was approximated to the volume of a 1‐yuan coin, 15 mL to a tablespoon, 300 mL to a rice bowl and 500 mL to a bottle of mineral water. Patients were questioned regarding symptoms suggestive of circulatory compromise, including dizziness, palpitations, visual darkening and syncope.

Based on the occurrence of delayed postoperative bleeding, patients were categorised into a bleeding group (case group) and a nonbleeding group (control group).

#### 2.5.5. Short‐Term Follow‐up

All patients were followed up at 1 week and 1 month postoperatively, either in the clinic or by telephone. Based on the descriptions and photographs of the patient, the presence or absence of bleeding, as well as the timing and volume of bleeding, was recorded.

#### 2.5.6. Long‐Term Follow‐Up

At 2 years postoperatively, clinical effectiveness was assessed by telephone follow‐up and either digital rectal examination or repeat colonoscopy. Outcomes were defined as follows: cure, complete resolution of haemorrhoids and associated symptoms; effective, reduction in haemorrhoids and associated symptoms; and ineffective, no improvement or worsening of haemorrhoids and related symptoms. The total effective rate was calculated as the sum of cured and effective cases.

### 2.6. Statistical Analysis

Statistical analysis was performed using SPSS software (Version 27.0; IBM, Armonk, New York, United States). Categorical variables are presented as frequencies and percentages (%). Univariate logistic regression analysis was conducted to identify potential factors associated with delayed bleeding. Variables with *p* values < 0.05 in the univariate analysis were entered into the multivariate logistic regression model. The odds ratios (ORs) and 95% confidence intervals (CIs) were calculated to determine independent risk factors. A *p* value < 0.05 was considered statistically significant. The statistical analysis was reviewed by a biomedical statistician.

## 3. Results

### 3.1. Clinical Characteristics

**FIGURE 2:**
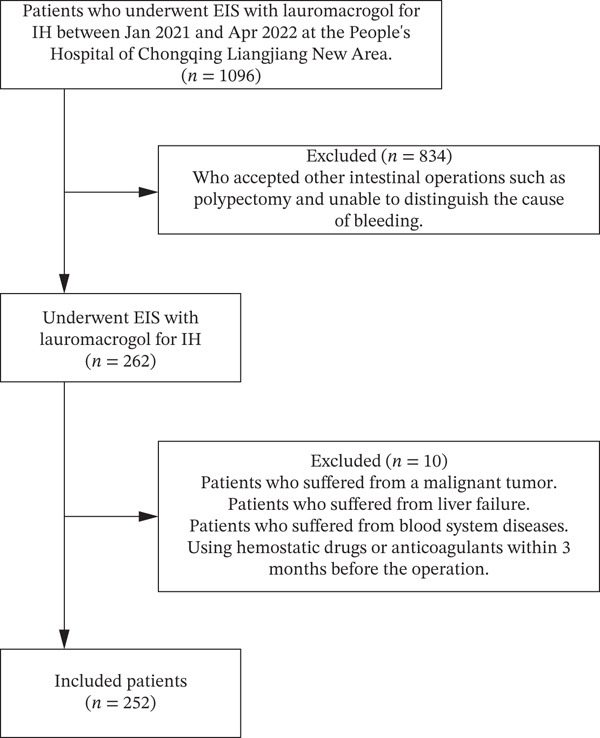
Patient screening flowchart. EIS, endoscopic injection sclerotherapy; IH, internal haemorrhoids.

From January 2021 to April 2022, 1096 patients underwent EIS for IH at our hospital. The patient screening process is illustrated in Figure 2. We excluded 834 patients who underwent additional intestinal procedures, such as polypectomy, making it difficult to determine the source of bleeding, and 10 patients with malignant tumours, liver failure, haematological disorders or use of haemostatic or anticoagulant agents within 3 months prior to surgery. Ultimately, 252 patients were included in the study, comprising 119 male and 133 female individuals, with a mean age of 46.5 ± 13.3 years.

Overall, of 252 patients, 25 (9.9%), 207 (82.2%) and 20 (7.9%) had Goligher Grade I, II and III haemorrhoids, respectively. Bleeding was the predominant symptom in 190 patients (75.4%), anal prolapse and swelling in 49 (19.4%) and anal itching with mucous discharge in 13 (5.2%).

**FIGURE 3:**
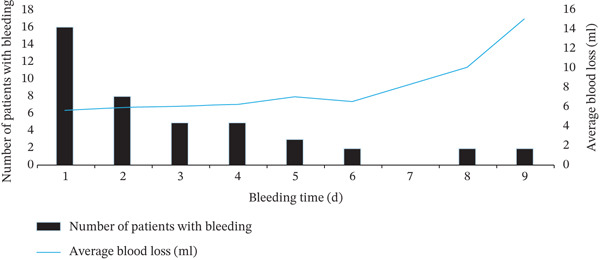
Distribution of delayed bleeding times and volumes.

Of 252 patients, 44 (17.5%) developed delayed haemorrhage after EIS. The median time to haemorrhage was 2 (range, 1–17) days. Delayed bleeding occurred in 16, 8, 5, 5, 3, 2, 0, 2 and 2 patients on postoperative Days 1 to 9, respectively. The distribution of bleeding onset and mean bleeding volume is shown in Figure 3. Overall, 43 patients (97.7%) experienced bleeding within 9 days postoperatively, whereas one patient developed bleeding on postoperative Day 17.

The mean volume of delayed bleeding among affected patients was 17.75 mL. Thirty‐nine patients (88.6%) had bleeding volumes < 10 mL, and 43 (97.7%) had volumes < 20 mL. The patient who bled on postoperative Day 17 experienced a bleeding volume of up to 500 mL.

### 3.2. Correlation Between Different Clinical Factors and Delayed Bleeding

Data were stratified into subgroups according to clinical variables and relevant clinical reference standards. The frequency and incidence of bleeding were calculated for each subgroup. Univariate logistic regression analysis was performed to identify factors associated with delayed bleeding (Table 1).

**TABLE 1:** Single‐factor logistic regression analysis of risk factors.

	Incidence of delayed haemorrhage	OR	95% CI	*p*value
Patient‐related factors				
Age (years)				
≤ 60	33/214 (15.42%)	Ref.		
> 60	11/38 (28.95%)	2.235	1.010–4.939	0.047
Sex				
Female	22/133 (16.54%)	Ref.		
Male	22/119 (18.49%)	1.144	0.596–2.193	0.685
Laboratory testing				
WBC (∗10^9^/L)				
Normal (3.5–9.5)	42/238 (17.65%)	Ref.		
Decreased (< 3.5)	1/12 (8.33%)	0.667	0.079–5.563	0.708
Increased (> 9.5)	½ (50.00%)	0.933	0.106–8.196	0.950
PLT (∗10^9^/L)				
Normal (125–350)	40/242 (16.53%)	Ref.		
Decreased (< 125)	3/9 (33.33%)	2.525	0.606–10.517	0.203
Increased (> 350)	1/1 (100.00%)	/	/	1.000
HGB (g/L)				
Decreased (< 115)	5/15 (33.33%)	Ref.		
Normal (115–150)	37/222 (16.67%)	0.379	0.122–1.174	0.093
Increased (> 150)	2/5 (40.00%)	1.333	0.165–10.742	0.787
ALB (g/L)				
Normal (40–55)	32/220 (14.55%)	Ref.		
Decreased (< 40)	11/32 (34.38%)	4.000	1.706–9.376	0.001
PT/s				
Normal (11–14.5)	44/205 (21.46%)	Ref.		
Extension (> 14.5)	0/7 (0.00%)	/	/	0.999
APTT/s				
Normal (28–43.5)	42/244 (17.21%)	Ref.		
Extension (> 43.5)	2/8 (25.00%)	1.603	0.312–8.218	0.571
TC (nmol/L)				
≤ 5.2	25/180 (13.89%)	Ref.		
> 5.2	19/72 (26.39%)	2.223	1.134–4.357	0.020
TG (nmol/L)				
Normal (0–1.7)	26/193 (13.47%)	Ref.		
Increased (> 1.7)	18/59 (30.51%)	2.820	1.412–5.629	0.003
LDLC (nmol/L)				
≤ 3.4	35/210 (16.67%)	Ref.		
> 3.4	9/42 (21.43%)	1.364	0.600–3.101	0.459
HDLC (nmol/L)				
Decreased (< 0.9)	5/28 (17.86)	Ref.		
Normal (0.9–1.94)	37/217 (17.05%)	0.946	0.337–2.648	0.915
Increased (> 1.94)	2/7 (28.57%)	1.840	0.274–12.348	0.530
Comorbidity				
Hypertension				
No	38/224 (16.96%)	Ref.		
Yes	6/28 (21.43%)	1.335	0.507–3.513	0.559
Diabetes				
No	42/240 (17.50%)	Ref.		
Yes	2/12 (16.67%)	0.943	0.199–4.461	0.941
Drug combination				
Carbazochrome sodium sulfonate				
No	43/228 (18.86%)	Ref.		
Yes	1/24 (4.17%)	0.187	0.024–1.423	0.105
Cefuroxime sodium				
No	36/206 (17.48%)	Ref.		
Yes	8/46 (17.39%)	0.994	0.427–2.309	0.989
Levofloxacin				
No	36/212 (16.98%)	Ref.		
Yes	8/40 (20.00%)	1.222	0.520–2.870	0.645
Indomethacin–furazolidone suppository				
No	27/176 (15.34%)	Ref.		
Yes	17/76 (22.37%)	1.590	0.807–3.131	0.180
Diosmin				
No	34/198 (17.17%)	Ref.		
Yes	10/54 (18.52%)	1.096	0.502–2.390	0.817
Fasting time				
24 h	36/197 (18.27%)	Ref.		
48 h	8/55 (14.55%)	0.761	0.331–1.749	0.521
WCS				
8 ≤ WCS < 15	7/111 (6.31%)	Ref.		
≥ 15	37/141 (26.24%)	5.286	2.254–12.396	< 0.001
Haemorrhoid‐related factors				
Goligher Grade				
I	3/25 (12.00%)	Ref.		
II	38/207 (18.36%)	1.649	0.469–5.793	0.435
III	3/20 (15.00%)	1.294	0.231–7.233	0.769
Injection points				
≤ 4	6/66 (9.09%)	Ref.		
> 4	38/186 (20.40%)	2.568	1.031–6.389	0.043
Injection dose/mL				
≤ 5	29/172 (16.86%)	Ref.		
> 5	15/80 (18.75%)	1.137	0.571–2.266	0.713

Abbreviations: ALB, albumin; APTT, activated partial thromboplastin time; 95% CI, 95% confidence interval differences; HDLC, high‐density lipoprotein cholesterol; HGB, haemoglobin count; LDLC, low‐density lipoprotein cholesterol; OR, odds ratio; PLT, platelet count; PT, prothrombin time; Ref., Reference; TC, total cholesterol; TG, triglyceride; WBC, white blood cells; WCS, Wexner Constipation Score.

The following variables were statistically significant (*p* < 0.05): age, albumin level (ALB), total cholesterol level (TC), triglyceride level (TG), postoperative WCS and number of injection sites. Specifically, age > 60 years (OR: 2.235, 95% CI: 1.010–4.939, *p* = 0.047), ALB < 40 g/L (OR: 4.000, 95% CI: 1.706–9.376, *p* = 0.001), TC > 5.2 mmol/L (OR: 2.223, 95% CI: 1.134–4.357, *p* = 0.020), TG > 1.7 mmol/L (OR: 2.280, 95% CI: 1.412–5.629, *p* = 0.003), postoperative WCS ≥ 15 (OR: 5.286, 95% CI: 2.254–12.396, *p* < 0.001) and > 4 injection sites (OR: 2.568, 95% CI: 1.031–6.389, *p* = 0.043) were identified as potential risk factors for delayed bleeding after EIS with lauromacrogol for IH.

Sex, comorbidities, routine haematological parameters, renal function, coagulation function, haemorrhoid grade, total injection volume, postoperative fasting duration and the use of the aforementioned medications during the perioperative period were not significantly associated with delayed bleeding.

### 3.3. Risk Factors for Delayed Haemorrhage

Variables with *p* < 0.05 in the univariate logistic regression analysis were entered into the multivariate logistic regression model, and the results are presented in Table 2.

**TABLE 2:** Multivariate logistic regression analysis of risk factors.

	OR	95% CI	*p*value
Age (years)			
≤ 60	Ref.		
> 60	2.123	0.847–5.320	0.108
TC (nmol/L)			
≤ 5.2	Ref.		
> 5.2	2.086	0.920–4.729	0.078
ALB (g/L)			
Normal (40–55)	Ref.		
Decreased (< 40)	5.093	1.927–13.461	0.001
TG (nmol/L)			
Normal (0–1.7)	Ref.		
Increased (> 1.7)	3.814	1.738‐8.368	< 0.001
WCS			
8 ≤ WCS < 15	Ref.		
≥ 15	5.34	2.142–13.315	< 0.001
Injection points			
≤ 4	Ref.		
> 4	4.425	1.576–12.422	0.005

Abbreviations: ALB, albumin; 95% CI, 95% confidence interval; OR, odds ratio; TC, total cholesterol; TG, triglyceride; WCS, Wexner Constipation Score.

Multivariate analysis demonstrated that ALB, TG, postoperative WCS score and number of injection sites remained statistically significant (*p* < 0.05). Specifically, ALB < 40 g/L (OR: 5.093, 95% CI: 1.927–13.461, *p* < 0.001), TG > 1.7 mmol/L (OR: 3.814, 95% CI: 1.738–8.368, *p* < 0.001), postoperative WCS ≥ 15 (OR: 5.340, 95% CI: 2.142–13.315, *p* < 0.001) and > 4 injection sites (OR: 4.425, 95% CI: 1.576–12.422, *p* = 0.005) were identified as independent risk factors for delayed bleeding.

### 3.4. Impact on Long‐Term Efficacy

After a 2‐year follow‐up, among the 44 patients in the delayed bleeding group, 24 were classified as cured, 18 as effective and 2 as ineffective. The total number of effective cases (cured + effective) was 42, yielding a total effective rate of 95.5% (42/44).

Among the 208 patients without delayed bleeding, 166 were cured, 38 were effective and 4 were ineffective. The total number of effective cases was 204, corresponding to a total effective rate of 98.1% (204/208). No significant difference was observed in overall effectiveness between the two groups at the 2‐year follow‐up (*χ* ^2^ = 0.242, *p* = 0.622; Table 3).

**TABLE 3:** Comparison of 2‐year effectiveness between the two groups.

Group	*n*	Cure	Effective	Invalid	Total effective
Delayed bleeding	208	166 (79.81%)	38 (18.27%)	4 (19.23%)	204 (98.08%)
Nondelayed bleeding	44	24 (54.55%)	18 (40.91%)	2 (4.55%)	42 (95.45%)
*χ* ^2^					0.242
*p*					0.622

Notably, the patient who experienced 500 mL of bleeding on postoperative Day 17 achieved complete resolution of haemorrhoidal symptoms at the 2‐year follow‐up.

## 4. Discussion

EIS for IH has become a widely used treatment for patients with Goligher Grade I–III haemorrhoids in China. However, given its relatively recent adoption in clinical practice, further time and evidence are required to comprehensively evaluate its associated complications [3].

Therefore, we investigated the incidence, timing, bleeding volume, prognosis and risk factors of delayed bleeding following EIS with lauromacrogol for IH.

### 4.1. Discussion on Bleeding Rate

This study demonstrated a delayed bleeding incidence of 17.5% following EIS for IH using lauromacrogol, indicating that delayed bleeding is a relatively common postoperative complication. Previous studies have reported bleeding rates after EIS for IH ranging from 5% to 25% [33, 34], suggesting that our findings are consistent with existing literature. Notably, bleeding rates are higher when using lauromacrogol solution compared with lauromacrogol foam [35, 36]; our hospital exclusively employs the solution form. Evidence suggests that the use of transparent caps may reduce the risk of bleeding during sclerotherapy for IHs [10, 33]; however, no transparent caps were utilised in this study.

### 4.2. Discussion on Bleeding Time and Amount

This study showed that > 80% of delayed bleeding occurred within 5 days postoperatively, with a volume of < 10 mL. Moreover, > 95% of delayed bleeding occurred within 9 days postoperatively, with a volume of < 20 mL, indicating that most patients experience early‐onset, low‐volume delayed bleeding after EIS for IH and that the prognosis is generally favourable. Additionally, after a 2‐year follow‐up, no significant difference was observed in overall effectiveness between patients with or without delayed bleeding.

### 4.3. Discussion on Risk Factors for Bleeding

This study demonstrates that hypoproteinaemia, hypertriglyceridemia, postoperative constipation and an increased number of injection points are risk factors for delayed bleeding after EIS for IH. Elevated TGs can reduce the collagen‐synthesising function of fibroblasts in the wound and affect blood viscosity and wound healing [37]. ALB participates in the renewal of mucosal cell components, promoting the growth and repair of wound cells and facilitating healing [38]. When ALBs are low, wound repair is impaired, resulting in thinner, more fragile wounds that are prone to bleeding [39]. In addition, hypoproteinaemia may be caused by inflammation, which further impairs wound healing [40]. Cheng et al. [41] reported that hypoalbuminemia is a risk factor for early rebleeding following endoscopic haemostasis and proton pump inhibitor infusion for peptic ulcer bleeding.

Constipation increases the risk of haemorrhoids [42]. After EIS for IH, constipation can elevate venous pressure in the haemorrhoids [43]. Hard stools and prolonged defecation increase friction between the stool and the wound surface, raising the risk of bleeding. On follow‐up, some patients who did not have preoperative constipation developed constipation postoperatively. Therefore, monitoring defecation after the procedure is essential. Patients should be prescribed postoperative laxatives and receive dietary guidance to maintain smooth bowel movements and avoid prolonged defecation. If constipation occurs, prompt medical attention should be sought.

An increased number of injection points increases tissue damage and stimulation, thereby raising the risk of postoperative bleeding [7, 34]. Additionally, as the number of injection points increases, the total dose of sclerosing agents delivered into the blood vessels rises, increasing defecation resistance and friction at the wound site, further elevating the risk of bleeding [35, 36]. In this study, four patients with three injection points experienced no bleeding. However, when the number of injection points exceeds four, the risk of bleeding rises significantly, and further increases continue to elevate this risk. For patients requiring more injection points, injections should be administered in batches to reduce bleeding risk.

### 4.4. Case Discussion on Severe Bleeding

**FIGURE 4:**
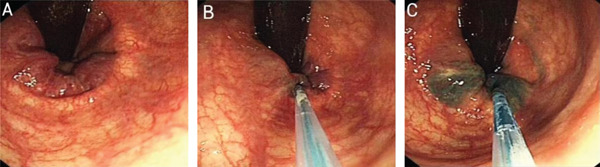
Endoscopic injection sclerotherapy in a patient who experienced 500 mL of delayed bleeding 17 days postprocedure. **(A)** Pre‐injection. Internal haemorrhoids and the anorectal line are exposed. **(B)** During injection. The injection distance is excessive, and the needle is extended too far, complicating observation and manipulation. The needle cannot be positioned close to the dentate line, resulting in an injection site that is too high above the anal line. **(C)** During injection. The injection angle is slightly increased, the needle extends too far and the injection is deeper, placing it in a region with larger blood vessels.

**FIGURE 5:**
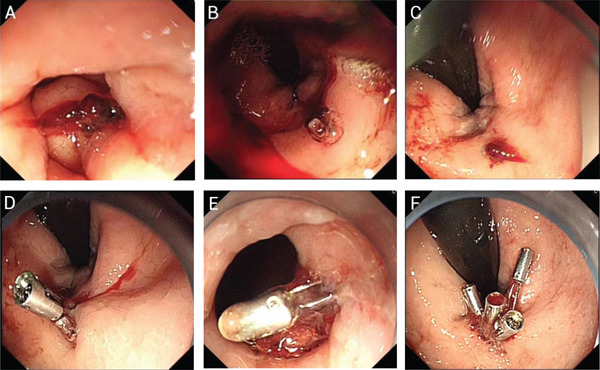
Endoscopic haemostasis for the patient who experienced 500 mL of delayed bleeding on the 17th day postsclerotherapy. **(A)** Blood clots adhere to the anorectal junction under forward endoscopy. **(B)** Blood obstructs the visual field under inverted endoscopy. **(C)** The field of view is cleared through repeated irrigation and suction, revealing a red blood clot above the anorectal line. **(D)** Haemostatic clips are applied, but the surrounding tissues are fibrotic and firm following sclerotherapy. The clips are applied superficially, and fresh bleeding persists after irrigation. **(E)** Forward endoscopy shows a shallowly positioned haemostatic clip, with an adjacent ulcer observed next to the bleeding site. **(F)** Bleeding is ultimately controlled using four haemostatic clips under inverted endoscopy, and no further bleeding is observed after irrigation.

The endoscopic image is shown in Figure 4. During the procedure, five injection sites were administered with a total of 4 mL of lauromacrogol. No bleeding occurred during EIS, and the patient was discharged without discomfort on 10 April 2022. However, she did not defecate for 5 days following discharge. On 15 April 2022, the patient experienced anal distension and frequent but unsuccessful attempts to pass stools. The outpatient department administered oral cathartics, prokinetic drugs, furazolidone–indomethacin suppositories and potassium permanganate sitz baths. The patient eventually defecated and discontinued the medications independently. A few days later, the patient developed a cold, with symptoms including sneezing, cough and diarrhoea. She sought treatment at a community clinic and received anti‐infective therapy, cough syrup and antidiarrhoeal medications, after which her symptoms improved, and she again discontinued treatment on her own. Subsequently, she developed constipation and resumed taking cathartic medications. On postoperative Day 17, the patient defecated five times, and haematochezia occurred following a severe coughing episode. She visited our emergency department and was admitted for hospitalisation. Despite administration of haemostatic drugs, bleeding continued, with a total volume of 500 mL. The life‐threatening situation required immediate endoscopic haemostasis. The haemostasis procedure under endoscopy is shown in Figure 5. Under endoscopy, a significant amount of blood was observed. After flushing, a thrombus head was identified above the anorectal line, with visible surrounding ulceration. The tissue, hardened by the previous EIS, was shallower after clamping with haemostatic clips. Four haemostatic clips were required to achieve successful haemostasis.

We analysed the direct cause of bleeding by reviewing the initial endoscopic images from the patient′s primary EIS (Figure 4). Following analysis and discussion, we considered that the injection distance may have been excessive, the injection needle may have been advanced too far, and the injection site may have been positioned too high above the anal line. We subsequently interviewed the operator who performed the EIS procedure. The operator recalled that potential contributing factors included an excessive injection volume at a single site, a high injection position and overly deep injection. The operator acknowledged that limited experience during the early stages of practice, together with suboptimal coordination between the operator and assistant, may have contributed to the complication. The operator had been practising gastroenterology for over 4 years and had performed more than 150 EIS procedures prior to treating this patient.

We speculated on the mechanism of bleeding in this patient as follows: On the one hand, an injection distance that is too great or an overly extended needle may result in an excessively deep injection, potentially damaging the intrinsic muscle layer or even deeper histological layers, leading to the formation of deep ulcers. In contrast, if the injection site is too far from the dentate line and even exceeds the anorectal line, larger and more abundant blood vessels, including the supplying artery, may be affected [44]. Stimulation by inducing factors, such as local infection, friction or increased intra‐abdominal pressure, may precipitate massive bleeding [45]. This case highlights the importance of avoiding excessively deep or vertical injections; the endoscope should be positioned at an appropriate distance to prevent being too far, and the depth and angle of the needle should be carefully controlled. The injection site should not be placed high above the anorectal line and may be slightly above the dentate line.

We further analysed systemic risk as follows: Technical aspects: This procedure employed an inverted‐view endoscope. Although the operator could clearly visualise the IHs using inverted endoscopy, precise control of the angle, depth and placement of the injection needle presented inherent challenges, relying heavily on the experience and tactile feedback of the surgeon. Team coordination: During inverted‐view injections, endoscopic manipulation (angle and field control) and injection delivery (depth and dose control) are performed separately by the operator and assistant. This case suggests that, during the initial phase of experience accumulation, coordination between the operator and assistant may be inconsistent, potentially leading to discrepancies between the intended injection site and the actual target. Equipment and protocol level: We did not routinely employ a transparent cap for assistance. Transparent caps stabilise the visual field and operative range, providing physical support for precise superficial injections. They are an important tool for preventing deep or misdirected injections. The 2025 Chinese Consensus [10] states that ‘endoscopic sclerotherapy requires transparent cap assistance’ and that ‘transparent caps should be used as appropriate for inverted endoscopy’. Our failure to utilise a transparent cap constituted a significant procedural risk factor.

Following this incident, we conducted a comprehensive review of relevant guidelines and technical procedures. We acknowledge that during the case accumulation phase of this study, our institution had not yet adopted transparent caps as standard equipment. This omission was partly because of limitations in available instrumentation and insufficient awareness of the latest technical protocols. At the outset of this research, we prioritised the superior overall visualisation of haemorrhoidal tissue afforded by inverted‐view endoscopy. This serious complication serves as a reminder of the necessity of adopting optimal and standardised technical approaches**.** With the publication of updated domestic and international guidelines and consensus statements in 2023 and 2025 (including the literature referenced), transparent cap‐assisted techniques have been strongly recommended because of their favourable safety profile. We have now begun implementing and mandating the use of transparent caps in our department. Professor Zhang Faming′s development of transparent cap‐assisted endoscopic EIS provides an effective strategy to prevent deep injections and severe complications [46]. We agree that this technique, by stabilising the mucosa with a transparent cap and providing a front‐facing injection perspective from within the anal canal (using a standard or modified inverted scope), enables the operator to maintain direct control over injection depth and position throughout the procedure. This substantially enhances procedural safety and precision and represents an optimal method for preventing deep injections. The core value of this technical evolution—from traditional inverted‐scope manoeuvres to transparent cap‐assisted precision injection—lies in shifting safety reliance from individual experience and tactile judgement to safeguards provided by standardised instruments and improved visualisation.

### 4.5. Discussion of Negative Results

This study further confirmed that perioperative administration of antibiotics, haemostatic agents, diosmin or furazolidone–indomethacin suppositories was not significantly associated with delayed postoperative haemorrhage. No current guidelines recommend the routine use of these medications [10, 46]. We acknowledge that such prescribing decisions were largely based on clinical experience. However, our literature review indicates that supporting evidence for these agents is limited. In future practice, we will adhere more strictly to guideline‐based and evidence‐based medicine principles and avoid unnecessary medication use. No significant difference was observed in the incidence of delayed postoperative haemorrhage between 24 and 48‐h postoperative fasting periods. Accordingly, we will avoid unnecessary prolongation of fasting duration in future practice.

### 4.6. Discussion on Limitations

This study had some limitations. The risk factor analysis could have included additional procedural factors, such as injection distance, depth, position and angle. However, the study had a retrospective design, and these operational details were difficult to quantify accurately. Consequently, we could not draw objective conclusions through statistical analysis of these injection factors. Moreover, the number of cases was small, and the retrospective design may have introduced recall bias. Finally, whether the patient experienced bleeding was determined through telephone or outpatient follow‐up, which may have introduced survey bias. Therefore, a prospective, large‐sample study with more objectively quantified data should be conducted [47].

## 5. Conclusions

In conclusion, delayed bleeding is a common postoperative complication of EIS with lauromacrogol for IH performed using the inverted colonoscope technique without transparent caps. Most cases involve small‐volume, early‐onset and self‐limited bleeding. Delayed postoperative bleeding after EIS does not affect long‐term efficacy. Identified risk factors for delayed bleeding include hypertriglyceridaemia, hypoproteinaemia, postoperative constipation and more than four injection sites.

During EIS for IHs using inverted colonoscopy without transparent caps, injection precision is of paramount importance. Operator inexperience, poor team coordination or procedural carelessness may result in incorrect injection depth, potentially leading to rare but serious delayed massive bleeding. Therefore, EIS should be performed with caution and in accordance with established guidelines.

## Author Contributions

Yue C. and Mingqiong W. contributed equally to this work; Yue C. and Mingqiong W. designed the research study, analysed the statistics and wrote the paper; Xing S., Yuxia Z., Mingke L., Peng P. and Ruizheng Z. contributed to the operations and provided clinical advice; Xing S. supervised the study; Yue C., Yuxia Z., Xing S., Mingke L., Ruizheng Z. and Chang L. contributed to data interpretation; Mingqiong W., Qin H. and Hongmei L. collected the data; Ruizheng Z. approved the final version of the manuscript for publication.

## Funding

No funding was received for this manuscript.

## Ethics Statement

This study was approved by the Institutional Review Board of our hospital.

## Consent

Patients signed informed consent forms to undergo endoscopy and sclerotherapy. During follow‐up, they were informed of their inclusion in a retrospective analysis and advised that the analysis used anonymous clinical data, and all patients provided verbal consent.

## Conflicts of Interest

The authors declare no conflicts of interest.

## Supporting information


**Supporting Information** Additional supporting information can be found online in the Supporting Information section. STROBE Statement The authors have read the STROBE Statement—checklist of items, and the manuscript was prepared and revised according to the STROBE Statement—checklist of items.

## Data Availability

No additional data are available.
